# Low-molecular-weight heparin for hip fracture patients treated with osteosynthesis: should thromboprophylaxis start before or after surgery? An observational study of 45,913 hip fractures reported to the Norwegian Hip Fracture Register

**DOI:** 10.1080/17453674.2018.1519101

**Published:** 2018-10-17

**Authors:** Sunniva Leer-Salvesen, Eva Dybvik, Lars B Engesaeter, Ola E Dahl, Jan-Erik Gjertsen

**Affiliations:** 1 Department of Clinical Medicine, University of Bergen, Bergen, Norway;; 2 Norwegian Arthroplasty Register, Department of Orthopaedic Surgery, Haukeland University Hospital, Bergen, Norway;; 3 Innlandet Hospital Trust, Elverum, Norway;; 4 Thrombosis Research Institute, London, UK

## Abstract

Background and purpose — Controversies exist regarding thromboprophylaxis in orthopedic surgery. We studied whether the thromboprophylaxis in hip fracture patients treated with osteosynthesis should start preoperatively or postoperatively. Data were extracted from the nationwide Norwegian Hip Fracture Register (NHFR). The risks of postoperative deaths, reoperations, and intraoperative bleeding were studied within 6 months after surgery.

Patients and methods — After each operation for hip fracture in Norway the surgeon reports information on the patient, the fracture, and the operation to the NHFR. Cox regression analyses were performed with adjustments for age group, ASA score, sex, duration of surgery, and year of surgery. During the period 2005–2016, 96,599 hip fractures were reported to the register. Only osteosyntheses where low-molecular-weight heparin (LMWH) were given and with known information on preoperative start of the prophylaxis were included in the analyses. Dalteparin and enoxaparin were used in 58% and 42% of the operations respectively (n = 45,913).

Results — Mortality (RR =1.01, 95% CI 0.97–1.06) and risk of reoperation (RR =0.99, CI 0.90–1.08) were similar comparing preoperative and postoperative start of LMWH. Postoperative start reduced the risk of intraoperative bleeding complications compared with preoperative start (RR =0.67, CI 0.51–0.90).

Interpretation — The initiation of LMWH did not influence the mortality or the risk of reoperation in hip fracture patients treated with osteosynthesis. Postoperative start of LMWH could possibly decrease the risk of intraoperative bleeding.

Among elderly hip fracture patients, vascular events caused by thrombosis are common and the use of chemical thromboprophylaxis is a well-established routine in the management of these patients. On the other hand, the risk of intraoperative bleeding also represents a major concern that may increase both the duration of surgery and risk of reoperation, anemia and transfusions (Vera-Llonch et al. 2006). The potential complications following both bleeding and thromboembolic events can in turn prolong the need for hospitalization and rehabilitation.

The Norwegian Hip Fracture Register (NHFR) has collected nationwide information on hip fractures since 2005 (Gjertsen et al. 2008). In a previous article based on data from the NHFR we found that postoperative start of LMWH increased both mortality and risk of reoperation compared with preoperative start after femoral neck fractures treated with hemiarthroplasty (Leer-Salvesen et al. 2017). On the other hand, preoperative start of the thromboprophylaxis did not increase the risk of intraoperative bleeding complications or reoperation due to hematoma.

Knowledge concerning the administration of thromboprophylaxis in hip fracture patients treated with osteosynthesis is, on the contrary, still sparse. By using data in the nationwide NHFR we wanted to compare the effect of preoperative start versus postoperative start of thromboprophylaxis in hip fracture patients treated with osteosynthesis. Primary endpoints were mortality and reoperations in the first 6 months after surgery and intraoperative bleeding complications. Secondary endpoints were reoperations due to infection or hematoma.

## Patients and methods

The NHFR started registration of primary operations and reoperations for all hip fractures in Norway in 2005 (Gjertsen et al. 2008). Compared with the Norwegian Patient Registry, the completeness of primary operations in the NHFR is 88% for osteosyntheses (Furnes et al. 2018). After each operation the surgeon fills in a one-page paper form. The form includes information on age, sex, cognitive function, type of fracture (intracapsular femoral neck fractures classified as Garden 1–2 or 3–4; trochanteric fractures classified as 2-fragmented [AO/OTA A1], multi-fragmented [AO/OTA A2], or intertrochanteric [AO/OTA A3]; and subtrochanteric fractures), ASA class, and duration of surgery. The form further provides information on the chemical thromboprophylaxis given (if used or not, which drug, dosage, and whether the first dose was given preoperatively or postoperatively). The surgeon should also report intraoperative complications, including major bleeding complications, to the register. A reoperation was defined as any secondary surgery following the primary operation. The cause of reoperation is reported by the surgeon on a similar paper form to that used for the primary operation.

**Figure 1.**
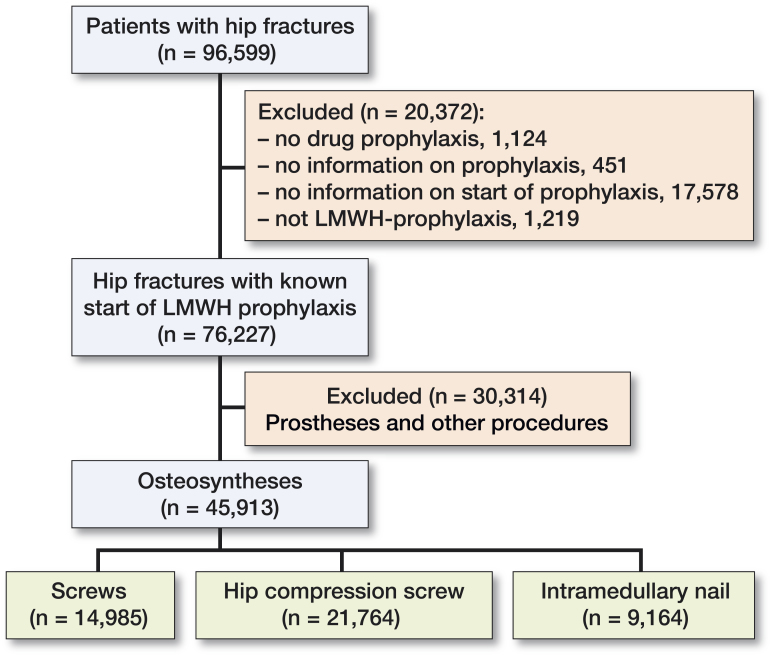
Flow chart for patients included in the study.

In the period 2005–2016, 96,599 primary operations for hip fractures were reported to the NHFR (Figure 1). 15 different types of drugs for prophylaxis had been used. However, low-molecular-weight heparin (LMWH) dominated entirely. To obtain a more homogeneous study group, operations with drugs other than LMWH were excluded. 45,913 patients operated with osteosynthesis were included. Dalteparin (Fragmin, Pfizer) was used in 58% (26,469 operations) and enoxaparin (Klexane, Sanofi-Aventis) was used in 42% (19,444 operations).

### Statistics

Survival analyses were performed using the Kaplan–Meier and Cox regression methods. Patients who died or emigrated during follow-up were identified from files provided by Statistics Norway, and the follow-up time for these patients was censored at the date of death or emigration. Only the first 6 postoperative months were included in the analyses as this period was considered most relevant in the present investigation.

The Cox multiple regression model was used to compare the relative risks of postoperative death and revision (failure-rate ratios) among patients where the thromboprophylaxis started preoperatively compared with postoperatively, with adjustments for possible influences of sex, duration of surgery, age of the patient at surgery (grouped in decades), ASA classification and year of surgery. Analyses on mortality and risk of reoperations within 7 days postoperatively, within 30 days postoperatively, and within 6 months postoperatively were performed. In addition to reoperations for any reason, we also investigated reoperations due to infection or hematoma and reported intraoperative bleeding complications. When investigating reoperations due to hematomas, patients with concurrent other causes for reoperation were excluded from the analyses.

95% confidence intervals (CIs) were calculated for relative risks. Number needed to harm (NNH) was calculated and defined as the number of patients treated with preoperative start of LMWH in order to cause 1 intraoperative bleeding complication because of preoperative start compared with postoperative start assuming a direct causal effect. The number needed to harm was calculated as an inverse value of the risk difference between the preoperative and postoperative start of LMWH.

#### Sub-analyses

The osteosyntheses were divided into 3 sub-groups to investigate the most frequently used surgical procedures: screw osteosynthesis (14,985 operations, 33%), hip compression screw (21,764 operations, 47%), and intramedullary nail (9,164 operations, 20%).

Further, sub-analyses were performed for ASA classes 1–2 and 3–5. Lastly, duration of surgery was investigated: Group 1 with a short duration of surgery (less than 16 minutes for screws, less than 40 minutes for nails or compression screws, 25% of the study populations), group 2 with a median duration of surgery (16–30 minutes for screws, 40–75 minutes for compression screws and 40–84 minutes for nails, 25–75% of the study populations), and group 3 with a long duration of surgery (more than 30 minutes for screws, more than 75 minutes for compression screws and more than 84 minutes for nails, 25% of the study populations).

Assessments of proportionality in the Cox models were performed using log minus log plots of the adjusted survival curves, and the proportionality assumptions were fulfilled. We used the statistical software packages IBM SPSS® Statistics, version 24.0 for Windows (IBM Corp, Armonk, NY, USA) and the statistical package R, version 3.4.0, (http://www.R-project.org), for the statistical analyses. P-values <0.05 were considered statistically significant.

### Ethics, funding, and potential conflicts of interest

The NHFR has permission from the Norwegian Data Inspectorate to collect patient data based on written consent from the patients. (Permission issued January 3, 2005; reference Number 2004/1658-2 SVE/-). Informed consent from patientsis entered in the medical records at each hospital. The Norwegian Hip Fracture Register is financed by the Western Norway. The authors declare no competing interests.

## Results

**Figure 2.**
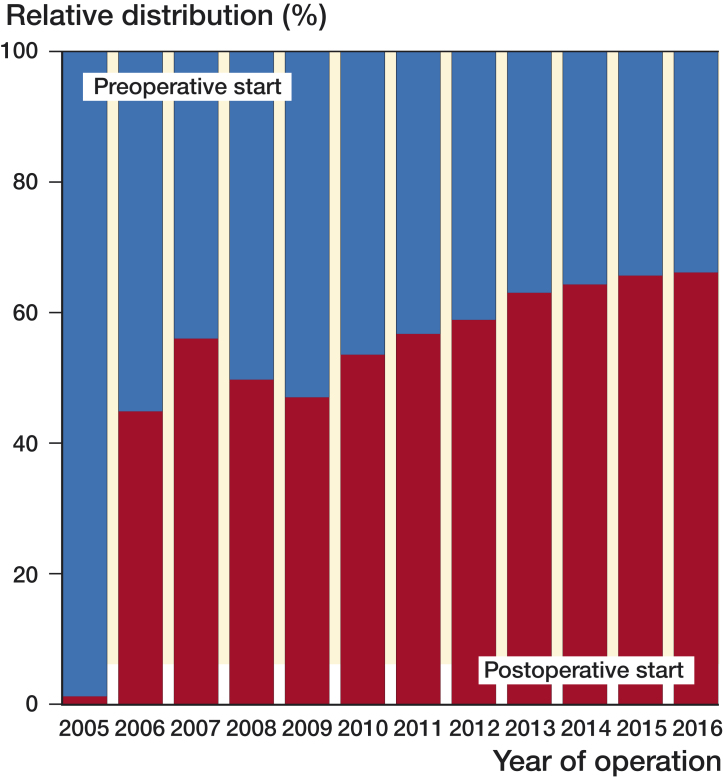
Timeline demonstrates the development in start of thromboprophylaxis from 2005 to 2016 for the patients observed in the study (n = 45,913). Hip fracture patients operated with osteosynthesis with known start of LMWH thromboprophylaxis (dalteparin or enoxaparin).

Table 1 presents baseline information on the patients included. Thromboprophylaxis was given preoperatively in 45% (20,563 operations) and postoperatively in 55% (25,350 operations). When comparing patients with a preoperative versus postoperative start of LMWH, no statistically significant differences in age, sex, comorbidity, or duration of surgery were found. There was an increase in postoperative initiation of LMWH during the studied period (Figure 2).

**Table 1.** Patients included in the study

	Start of prophylaxis	
Factor	Preoperative	Postoperative
Hip fractures with osteosynthesis, n (%)	20,563 (45)	25,350 (55)
Mean duration of surgery^a^	51 (33)	51 (34)
Mean age at fracture (years) (SD)	80 (12)	80 (13)
Women (%)	69	69
ASA groups, n (%)		
ASA 1	1,363 (6.6)	1,857 (7.3)
ASA 2	7,015 (34)	8,793 (35)
ASA 3	10,362 (50)	12,860 (51)
ASA 4	1,475 (7)	1,520 (6)
ASA 5	34 (0.2)	27 (0.1)
Missing	314 (1.5)	293 (1.2)
Type of surgery^b^		
Screws, n (%)	6,781 (45)	8,204 (55)
Mean duration of surgery^a^	26 (14)	26(14)
Hip compression screw, n (%)	9,939 (46)	11,825 (54)
Mean duration of surgery^a^	62 (31)	61 (30)
Intramedullary nail, n (%)	3,843 (42)	5,321 (58)
Mean duration of surgery^a^	66 (38)	66 (41)

a Values are minutes (SD)

b Screws include operations with Olmed screws. Hip compression screws include operations with a dynamic hip screw (DHS) with or without a support plate. Intramedullary nails include long and short nails with or without the use of an interlocking screw.

### Mortality

**Figure 3.**
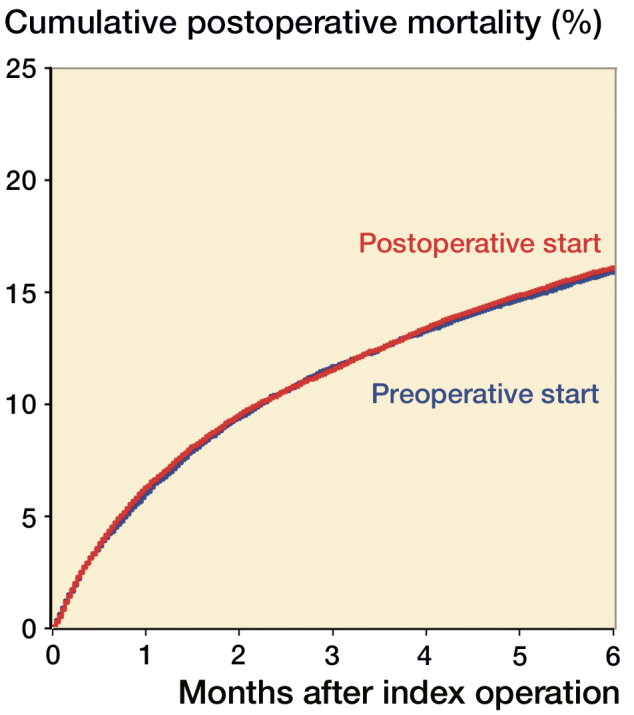
Postoperative mortality for hip fracture patients treated with osteosynthesis.

Overall mortality after 6 months was 19% (8,751 of 45,913 patients). No statistically significant difference in mortality between preoperative and postoperative start of LMWH could be found. The results were consistent after 7, 30, and 180 days (Table 2, Figure 3).

**Table 2.** Mortality and risk of reoperation 180 days postoperatively after osteosynthesis for hip fracture

		Start of prophylaxis, n (%)				
	Total, n (%)	Preoperative	Postoperative	RR^a^	95% CI	p-value
7 days postoperatively (n = 45,913)						
Mortality	1,050 (2.3)	481 (2.3)	569 (2.2)	1.02	0.90–1.16	0.7
Reoperations	190 (0.4)	76 (0.4)	114 (0.4)	1.18	0.88–1.58	0.3
Reoperation due to infection	5 (0.0)	1 (0.0)	4 (0.0)	3.50	0.38–32.0	0.3
Reoperation due to hematoma	9 (0.0)	3 (0.0)	6 (0.0)	1.86	0.46–7.49	0.4
30days postoperatively (n = 45,913)						
Mortality	3,534 (7.7)	1,606 (7.8)	1,928 (7.6)	1.05	0.98–1.12	0.2
Reoperations	627 (1.4)	275 (1.3)	352 (1.4)	1.12	0.95–1.32	0.2
Reoperation due to infection	66 (0.1)	30 (0.1)	36 (0.1)	0.88	0.54–1.44	0.6
Reoperation due to hematoma	18 (0.0)	6 (0.0)	12 (0.0)	2.04	0.71–5.82	0.2
180 days postoperatively (n = 45,913)						
Mortality	8,751 (19)	4,049 (20)	4,702 (19)	1.01	0.97–1.06	0.6
Reoperations	2,067 (4.5)	966 (4.7)	1,101 (4.3)	0.99	0.90–1.08	1.0
Reoperation due to infection	115 (0.3)	49 (0.2)	66 (0.3)	1.02	0.70–1.49	0.9
Reoperation due to hematoma	19 (0.0)	6 (0.0)	13 (0.1)	2.18	0.78–6.18	0.1

a Cox relative revision risk (RR) (with preoperative start of prophylaxis as reference) is given with adjustments for possible influences of sex, ASA class, age group of the patient at surgery, duration of surgery, and year of surgery.

### Reoperations

**Figure 4.**
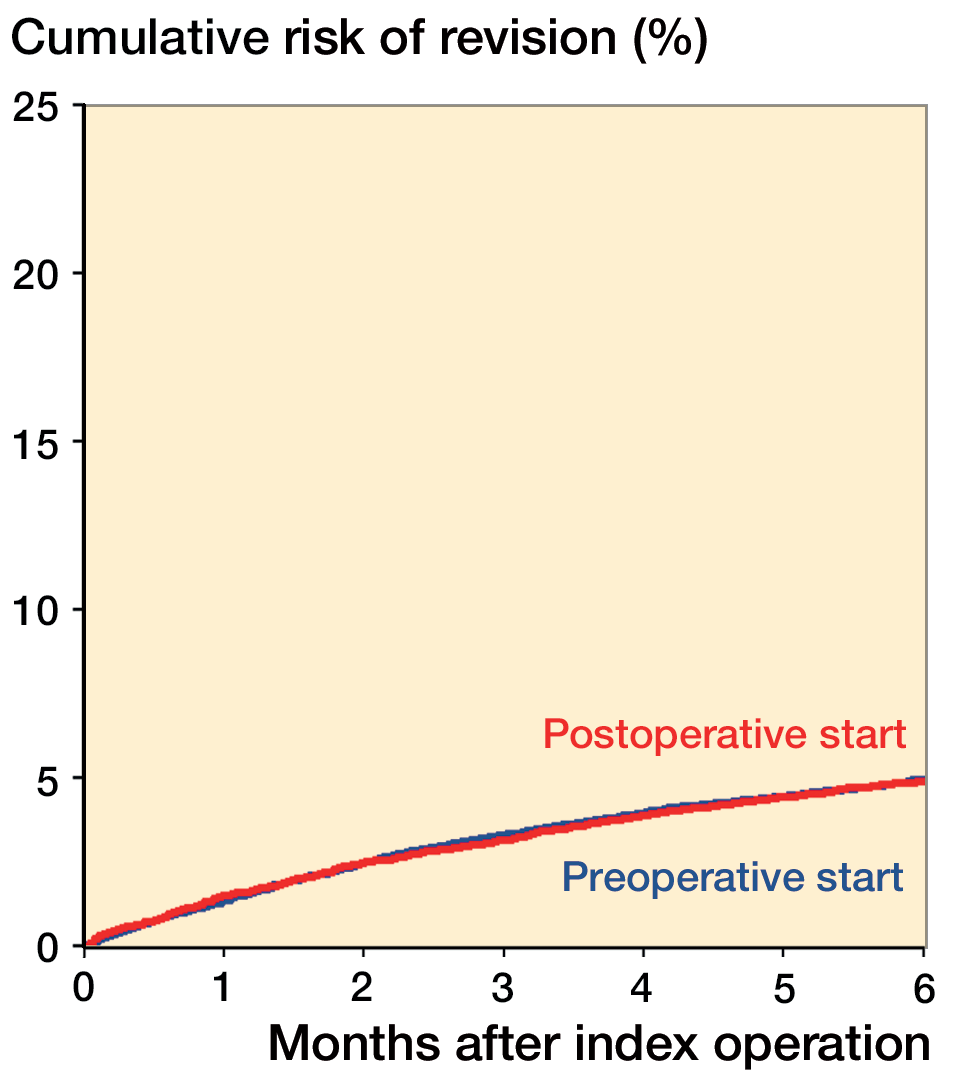
Risk of reoperation for hip fracture patients treated with osteosynthesis.

After 6 months 4.5% (2,067 of 45,913 operations) had been reoperated. There were 115 reoperations (0.3%) due to infection. Only 19 reoperations due to hematoma were reported to the register. No statistically significant differences in reoperations for any cause, reoperations due to infection, or reoperation due to hematoma could be found when comparing a preoperative versus postoperative start of thromboprophylaxis (Table 2, Figure 4).

### Intraoperative bleeding complications

1,294 (3.0%) intraoperative complications were reported after osteosyntheses. 208 (16% of all reported complications) were intraoperative bleedings. Postoperative start of LMWH decreased the risk of intraoperative bleeding complications compared with preoperative start (RR =0.67 (CI 0.51–0.90), NNH =434) (Table 3).

**Table 3.** Risk of intraoperative bleeding complications after osteosynthesis (n = 45,913) in hip fractures receiving screws (n = 14,985), hip compression screws (n = 21,764), or medullary nails (n = 9,164)

	Intraoperative bleeding, n (%)				Risk	Risk	
Treatment	Preop. start	Postop. start	RR^a^ (95% CI)	p-value	Preop.– Postop.	difference	NNH^b^
Osteosynthesis	118 (0.6)	90 (0.4)	0.67 (0.51–0.90)	0.007	0.0060 – 0.0037	0.0023	434
Screws	3 (0.0)	2 (0.0)	0.43 (0.07–2.68)	0.4	0.0004 – 0.0002	0.0002	4,940
HCS	102 (1.0)	71 (0.6)	0.64 (0.47–0.87)	0.004	0.0108 – 0.0063	0.0045	222
Intramedullary nail	13 (0.3)	17 (0.3)	1.06 (0.50–2.26)	0.9	0.0036 – 0.0034	0.0002	4,684

a See Table 2.

b NNH: Number of patients treated with preoperative start of LMWH in order to cause one intraoperative bleeding complication because of preoperative LMWH start compared with postoperative LMWH start if there is a direct causal effect. The NNH was calculated as an inverse value of the risk difference (RD) between the methods [1/(risk preoperative start–risk postoperative start)].

### Type of osteosynthesis

The hip fractures were reviewed in subgroups based on the type of osteosynthesis performed (Table 4). There was an increased 30-day mortality risk after operation with hip compression screw when LMWH was initiated postoperatively compared with preoperatively (RR =1.10; CI 1.00–1.21). For other types of osteosyntheses, the startup time of LMWH prophylaxis had no statistically significant influence on mortality 7, 30, or 180 days after surgery. After operation with intramedullary nail there was an increased 180-day risk of reoperation due to infection after postoperative start of thromboprophylaxis compared with preoperative start (RR =3.7; CI 1.04–13.2). No other statistically significant differences in risk for reoperation due to any cause, reoperation due to infection, or reoperation due to hematoma could be found (Table 4). After operation with hip compression screw there was a decreased risk of intraoperative bleeding complications after postoperative start of LMWH compared with preoperative start (RR =0.64; CI 0.47–0.87) (see Table 3).

**Table 4.** Mortality and risk of reoperation 180 days postoperatively after osteosynthesis in hip fractures receiving screws, hip compression screw, or medullary nails

		Start of prophylaxis, n (%)				
	Total, n (%)	Preoperative	Postoperative	RR^a^	95% CI	p-value
Screws (n = 14,985)						
Mortality	2,776 (19)	1,353 (21)	1,423 (18)	1.01	0.92–1.08	1.0
Reoperations	1,226 (8.2)	593 (9.1)	633 (8.0)	0.97	0.86–1.09	0.6
Reoperation due to infection	20 (0.1)	11 (0.2)	9 (0.1)	0.76	0.30–1.9	0.6
Reoperation due to hematoma	4 (0.0)	0 (0.0)	4 (0.1)	–	–	–
Hip compression screw (n = 21,764)						
Mortality	4,264 (20)	1,942 (20)	2,322 (20)	1.01	0.98–1.1	0.2
Reoperations	580 (2.7)	266 (2.7)	314 (2.7)	0.97	0.82–1.2	0.7
Reoperation due to infection	77 (0.4)	35 (0.4)	42 (0.4)	0.92	0.58–1.4	0.7
Reoperation due to hematoma	11 (0.1)	4 (0.0)	7 (0.1)	1.9	0.49–7.6	0.3
Intramedullary nail (n = 9,164)						
Mortality	1,711 (19)	754 (20)	957 (18)	0.96	0.87–1.1	0.4
Reoperations	261 (2.8)	107 (2.8)	154 (2.9)	1.2	0.91–1.5	0.2
Reoperation due to infection	18 (0.2)	3 (0.1)	15 (0.3)	3.7	1.04–13	0.04
Reoperation due to hematoma	4 (0.1)	2 (0.1)	2 (0.0)	1.2	0.14–9.8	0.9

a See Table 2.

### ASA classification

Patients were stratified into ASA classes 1–2 and ASA classes 3–5. For both subgroups, no statistically significant differences in mortality or risk of reoperation could be found within 180 days of follow-up between preoperative and postoperative start of LMWH (data not shown).

### Duration of surgery

Patients treated with intramedullary nail with long duration of surgery (> 84 minutes, upper quartile) had the most marked increased risk of reoperation due to infection after postoperative start of LMWH compared with preoperative start (RR =8.2; CI 1.03–65). The startup time of LMWH did not statistically significantly influence the risk of reoperation due to infection in patients treated with intramedullary nails with shorter operation time or for other osteosyntheses (data not shown).

For patients treated with hip compression screw the risk of intraoperative bleeding complication decreased after postoperative start of LMWH with long duration of surgery (> 75 minutes, upper quartile) (RR =0.68; CI 0.47–0.99). The startup time of LMWH did not influence the risk of intraoperative bleeding complication in patients treated with hip compression screw with shorter operation time (data not shown).

## Discussion

When comparing preoperative versus postoperative start of LMWH for hip fracture patients treated with osteosynthesis, no differences in mortality or risk of reoperation were found. Preoperative start of LMWH was found to give more intraoperative bleeding complications for patients treated with hip compression screws, but not for patients receiving intramedullary nails or screws.

Whether chemical prophylaxis should start preoperatively or postoperatively is controversial (Ettema et al. 2009, Borgen et al. 2013). In Europe the LMWH prophylaxis has traditionally started before hip fracture surgery (Ettema et al. 2009), while in North America a postoperative initiation has been common (Kearon and Hirsh 1995, Gomez-Outes et al. 2012, Lassen et al. 2012). This divergent practice between the continents may be continued based on traditional consequences. The fear of bleeding-related complications has been most critical for surgeons in North America due to medico-legal issues. In Europe, on the other hand, such complications have been the common responsibility of the department and the main focus has been to prevent local and systemic thromboembolic events. Due to the tremendous costs of antithrombotic drug development, several companies have recently developed a common regimen for both continents. Nevertheless, timing in relation to surgery has been considered important for the efficacy-to-safety balance in any pharmaceutical anticoagulant program. Trials funded by the industry have primarily focused on detecting thromboses with mandatory radiology following the surgical intervention. Unfortunately, some studies have been criticized for underestimating the challenges bleeding and wound complications can present following surgery (Parvizi et al. 2007, Lachiewicz 2009, Dahl et al. 2010). Second, the reported trials have been designed to show potential favorable effects of new experimental regimes versus established regimes (Yoshida et al. 2013). In contrast, our register study compares the same compounds when investigating preoperative versus postoperative start of thromboprophylaxis. To our knowledge, there no study of this size has been conducted investigating the startup time of thromboprophylaxis in hip fracture surgery.

When studying femoral neck fractures treated with hemiarthroplasty, preoperative start of thromboprophylaxis reduced mortality within 6 months of surgery (Leer-Salvesen et al. 2017). This favorable effect of preoperative LMWH administration was most pronounced in the first postoperative weeks. Nevertheless, the preoperative effect was also robust over time and no catch-up effect was noticed during 6 months of observation. In the present study investigating hip fractures treated with osteosynthesis, no such protective effect of preoperative start of LMWH could be detected, either for the whole group or for subgroups of patients receiving screw osteosynthesis, hip compression screw, or intramedullary nail. In the frail elderly undergoing hip fracture, the primary trauma and subsequent surgery have been shown to significantly impact the immediate and long-term mortality (Talsnes et al. 2011, 2013). As shown in our previous study, preoperative administration of LMWH may contribute to reducing mortality following hemiarthroplasty (Leer-Salvesen et al. 2017). Importantly, osteosynthesis for hip fractures seem to induce less trauma and thrombin-driven vascular complications as compared with surgery with hemiprosthesis. Thus, the start of prophylaxis in relation to surgery might be less important when conducting osteosynthesis procedures.

Preoperative start of thromboprophylaxis did not increase the risk of reoperation after osteosyntheses compared with postoperative start. In previous discussions, the risk of reoperation has in particular been brought forward as an argument to start thromboprophylaxis postoperatively (Lassen et al. 2012). This argument has partly been based on the fear of intraoperative bleeding complicating the surgical intervention. The fear of bleeding might also explain the gradual shift from preoperative to postoperative initiation of LMWH observed during the last decade. Relevantly, our study did demonstrate a decreased risk of intraoperative bleeding when the LMWH was initiated postoperatively compared with preoperatively for patients receiving osteosynthesis. In contrast, we did not find a decreased risk of intraoperative bleeding when the LMWH was initiated postoperatively in femoral neck fractures treated with hemiarthroplasty (Leer-Salvesen et al. 2017).

Postoperative start of LMWH decreased the risk of intraoperative bleeding in connection with hip compression screws. However, in patients operated with screw osteosynthesis or intramedullary nail a postoperative start of LMWH did not influence the risk of intraoperative bleeding. Screw osteosynthesis and intramedullary nail for hip fractures are most often performed as mini-invasive surgery, which may explain why risk of intraoperative bleeding complications is not affected by LMWH. Since displaced femoral neck fractures in the elderly are most often treated with an arthroplasty (Gjertsen et al. 2017), patients treated with screw osteosynthesis are younger than patients treated with osteosynthesis for extracapsular hip fractures. Less preoperative bleeding and younger age might explain why the start of LMWH administration does not significantly affect the risk of mortality, reoperation, or bleeding when using screw fixation.

Extracapsular hip fractures may be treated with hip compression screw or intramedullary nail. The complexity of the fracture will affect the risks of infection, bleeding, and reoperation. Intramedullary nails are increasingly used for complex trochanteric and subtrochanteric fractures. Postoperative start of LMWH compared with preoperative start increased the risk of reoperations due to infection after intramedullary nails. Hip fractures with time-consuming intramedullary nailing (more than 84 minutes, upper quartile) had a more than 8 times increased risk of reoperation due to infection after postoperative start of LMWH compared with preoperative start. A possible explanation could be that preoperative start of thromboprophylaxis induces bleeding earlier, which allows hemostasis or hematoma evacuation during surgery. Contrariwise, postoperative start of thromboprophylaxis LMWH may postpone traumatic bleeding and produce a late hematoma, which predispose to infection. A longer duration of surgery is often related to complex fractures, other concurrent intraoperative complications, or less experienced surgeons. These factors may also influence the risk of infection and potentiate the protective effect of a preoperative LMWH. However, due to limited number of patients in the sub-studies the results may be interpreted with caution.

### Strength and limitations

Our study is not a randomized controlled study (RCT) and may consequently be categorized as a hypothesis-creating study. However, when examining rare adverse events and large patient numbers, observational register studies remain an achievable method compared with RCTs. Nevertheless, this is, to our knowledge, the first study of its kind comparing preoperative versus postoperative benefit of the same compounds. The strength in our study is the inclusion of data from all surgical units treating hip fractures in one country. Accordingly, the external validity of the results is high. The inclusion of multiple hospitals may influenceour results. However, as we believe each hospital has routines in thromboprophylaxis administration, the inclusion of all hospitals has not been used as a confounder in our analyses. The register is dependent on volunteer reporting from individual surgeons. According to earlier coverage analyses, reoperations are more frequently left unreported to the register than primary operations, and such a tendency may affect our endpoints (Wiik et al 2014). It is, however, unlikely that differences in the reporting of reoperations after preoperative versus postoperative initiation of LMWH exist. Data concerning the start of LMWH are entered immediately after surgery by the responsible surgeon. Unfortunately, the NHFR only receives limited information concerning the usage of thromboprophylaxis in treatment of hip fractures. The surgeons do not report the exact moment in time of LMWH administration before and after surgery. Therefore, consequences of preoperative versus postoperative LMWH startup regimes are still partly unknown. The exact duration of LMWH treatment may not always be possible to predict immediately after surgery as later events or complications may alter the originally planned duration of treatment. Furthermore, after each operation the surgeon has to interpret whether the intraoperative bleeding was so extensive that it should be reported as a complication. Therefore, the findings regarding intraoperative bleeding must be interpreted with caution. Even though the study has weaknesses, the results are based on a large number of patients as well as consistent reporting of LMWH initiation to the register.

## Conclusion

Our data strongly indicate that preoperative compared with postoperative administration of LMWH does not influence mortality or risk of reoperation in hip fracture patients treated with osteosynthesis. However, postoperative start of LMWH does decrease the risk of reported intraoperative bleeding complications for operations with hip compression screw, but not with intramedullary nail or screw osteosynthesis.

The loyal reporting from all orthopedic surgeons made our studies possible.

The manuscript was produced by close teamwork between all authors. SLS and ED performed the statistical analyses. All authors contributed to the study design and interpretation of results.

*Acta* thanks Gerold Labek and other anonymous reviewers for help with peer review of this study.
